# Supplementary material for: HIVprotI: an integrated web based platform for prediction and design of HIV proteins inhibitors
Source: J Cheminform. 2018 Mar 9;10:12. doi: 10.1186/s13321-018-0266-y (PMC5845081; doi:10.1186/s13321-018-0266-y)
Supplement: Supplementary file 2 — Additional file 2. Source code of HIVProtI web server. [file 13321_2018_266_MOESM2_ESM.zip › HIVprotI_Source-code/hivprotI/weka-3-6-6/doc/weka/core/CheckGOE.html]

CheckGOE


---


|  |  |  |
| --- | --- | --- |
| **Overview** \| **Package** \| **Class** \| **Tree** \| **Deprecated** \| **Index** \| **Help** \| **Weka's home** | |  |
| **PREV CLASS**   **NEXT CLASS** | **FRAMES**    **NO FRAMES**     **All Classes** |  |
| SUMMARY: NESTED \| FIELD \| CONSTR \| METHOD | DETAIL: FIELD \| CONSTR \| METHOD |  |


---


## weka.core Class CheckGOE

```
java.lang.Object
  weka.core.Check
      weka.core.CheckGOE
```

**All Implemented Interfaces:**: OptionHandler, RevisionHandler

---

``` public class CheckGOE extends Check ```

Simple command line checking of classes that are editable in the GOE.

Usage:

 `CheckGOE -W classname -- test options` 


Valid options are:

```
 -D
  Turn on debugging output.
```

```
 -S
  Silent mode - prints nothing to stdout.
```

```
 -ignored <comma-separated list of properties>
  Skipped properties.
  (default: capabilities,options)
```

```
 -W
  Full name of the class analysed.
  eg: weka.classifiers.rules.ZeroR
  (default weka.classifiers.rules.ZeroR)
```

**Version:**
:   $Revision: 1.4 $

**Author:**
:   FracPete (fracpete at waikato dot ac dot nz)

---

**Constructor Summary**

`CheckGOE()`
  
          default constructor


| **Method Summary** | |
| --- | --- |
| `boolean` | `checkGlobalInfo()`             checks whether the object declares a globalInfo method. |
| `boolean` | `checkToolTips()`             checks whether the object declares tip text method for all its properties. |
| `void` | `doTests()`             Runs some diagnostic tests on the object. |
| `java.lang.String` | `getIgnoredProperties()`             Get the ignored properties used in checkToolTips() as comma-separated list (sorted). |
| `java.lang.Object` | `getObject()`             Get the object used in the tests. |
| `java.lang.String[]` | `getOptions()`             Gets the current settings of the object. |
| `java.lang.String` | `getRevision()`             Returns the revision string. |
| `boolean` | `getSuccess()`             returns the success of the tests |
| `java.util.Enumeration` | `listOptions()`             Returns an enumeration describing the available options. |
| `static void` | `main(java.lang.String[] args)`             Main method for using the CheckGOE. |
| `void` | `setIgnoredProperties(java.lang.String value)`             Sets the properties to ignore in checkToolTips(). |
| `void` | `setObject(java.lang.Object value)`             Set the object to work on.. |
| `void` | `setOptions(java.lang.String[] options)`             Parses a given list of options. |

**Methods inherited from class weka.core.Check**

`getDebug, getSilent, setDebug, setSilent`

**Methods inherited from class java.lang.Object**

`equals, getClass, hashCode, notify, notifyAll, toString, wait, wait, wait`

**Constructor Detail**

### CheckGOE

```
public CheckGOE()
```

:   default constructor


**Method Detail**

### listOptions

```
public java.util.Enumeration listOptions()
```

:   Returns an enumeration describing the available options.

    :   **Specified by:**: `listOptions` in interface `OptionHandler` **Overrides:**: `listOptions` in class `Check`
    :   **Returns:**: an enumeration of all the available options.

---


### setOptions

```
public void setOptions(java.lang.String[] options)
                throws java.lang.Exception
```

:   Parses a given list of options.


    Valid options are:

    ```
     -D
      Turn on debugging output.
    ```

    ```
     -S
      Silent mode - prints nothing to stdout.
    ```

    ```
     -ignored <comma-separated list of properties>
      Skipped properties.
      (default: capabilities,options)
    ```

    ```
     -W
      Full name of the class analysed.
      eg: weka.classifiers.rules.ZeroR
      (default weka.classifiers.rules.ZeroR)
    ```

    :   **Specified by:**: `setOptions` in interface `OptionHandler` **Overrides:**: `setOptions` in class `Check`
    :   **Parameters:**: `options` - the list of options as an array of strings **Throws:**: `java.lang.Exception` - if an option is not supported

---


### getOptions

```
public java.lang.String[] getOptions()
```

:   Gets the current settings of the object.

    :   **Specified by:**: `getOptions` in interface `OptionHandler` **Overrides:**: `getOptions` in class `Check`
    :   **Returns:**: an array of strings suitable for passing to setOptions

---


### setObject

```
public void setObject(java.lang.Object value)
```

:   Set the object to work on..

    :   **Parameters:**: `value` - the object to use.

---


### getObject

```
public java.lang.Object getObject()
```

:   Get the object used in the tests.

    :   **Returns:**: the object used in the tests.

---


### setIgnoredProperties

```
public void setIgnoredProperties(java.lang.String value)
```

:   Sets the properties to ignore in checkToolTips(). Comma-separated list.

    :   **Parameters:**: `value` - the list of properties **See Also:**: `checkToolTips()`

---


### getIgnoredProperties

```
public java.lang.String getIgnoredProperties()
```

:   Get the ignored properties used in checkToolTips() as comma-separated
    list (sorted).

    :   **Returns:**: the ignored properties **See Also:**: `checkToolTips()`

---


### getSuccess

```
public boolean getSuccess()
```

:   returns the success of the tests

    :   **Returns:**: true if the tests were successful

---


### checkGlobalInfo

```
public boolean checkGlobalInfo()
```

:   checks whether the object declares a globalInfo method.

    :   **Returns:**: true if the test was passed

---


### checkToolTips

```
public boolean checkToolTips()
```

:   checks whether the object declares tip text method for all its
    properties.

    :   **Returns:**: true if the test was passed

---


### doTests

```
public void doTests()
```

:   Runs some diagnostic tests on the object. Output is
    printed to System.out (if not silent).

    :   **Specified by:**: `doTests` in class `Check`

---


### getRevision

```
public java.lang.String getRevision()
```

:   Returns the revision string.

    :   **Returns:**: the revision

---


### main

```
public static void main(java.lang.String[] args)
```

:   Main method for using the CheckGOE.

    :   **Parameters:**: `args` - the options to the CheckGOE


---


|  |  |  |
| --- | --- | --- |
| **Overview** \| **Package** \| **Class** \| **Tree** \| **Deprecated** \| **Index** \| **Help** \| **Weka's home** | |  |
| **PREV CLASS**   **NEXT CLASS** | **FRAMES**    **NO FRAMES**     **All Classes** |  |
| SUMMARY: NESTED \| FIELD \| CONSTR \| METHOD | DETAIL: FIELD \| CONSTR \| METHOD |  |


---
